# Fulminant Anti-Neutrophil Cytoplasmic Antibody-Associated Vasculitis After 10 Years of Hydralazine Use

**DOI:** 10.7759/cureus.18974

**Published:** 2021-10-22

**Authors:** Jose R Russe-Russe, James R Pellegrini Jr., Alejandro Alvarez-Betancourt, Rezwan F Munshi, Prachi Anand

**Affiliations:** 1 Internal Medicine, Nassau University Medical Center, East Meadow, USA; 2 Rheumatology, Nassau University Medical Center, East Meadow, USA

**Keywords:** pauci-immune anti-neutrophil cytoplasmic antibody (anca)-associated vasculitis (aav), mpo-anca, antineutrophil cytoplasmic antibody (anca) associated vasculitis (aav), hydralazine-induced diffuse alveolar hemorrhage, hydralazine-induced vasculitis

## Abstract

Vasculitis, by definition, causes changes in the walls of blood vessels, including thickening, weakening, narrowing, and scarring, leading to inflammation and necrosis of the blood vessel walls. Small-vessel vasculitis is commonly associated with anti-neutrophil cytoplasmic antibodies (ANCA), which activate cytokine-primed neutrophils and monocytes that express ANCA antigens proteinase 3 (PR3) and myeloperoxidase (MPO) on their surface. The continuous injury and inflammation of these small vessels characterized by circulating immune complexes and antinuclear antibodies result in clinical features standard in all types of vasculitis. When a 59-year-old male with a history of heart failure, hypertension (on hydralazine 100 mg every eight hours for more than ten years), diabetes mellitus, and dyslipidemia presented to the hospital, he was complaining of hematuria, intermittent periumbilical abdominal pain, and 40-lb weight loss over four months. Initial evaluation showed symptomatic anemia and large blood cells with proteinuria on urine analysis. During his clinical course, the patient developed a new diffuse purpuric rash. Imaging showed systemic involvement with ground-glass opacities, diffuse alveolar hemorrhage, and peripancreatic inflammatory changes, consistent with small-vessel vasculitis. Immunological tests confirmed ANCA-associated vasculitis, and kidney biopsy showed ANCA-mediated pauci-immune glomerulonephritis supported by the salvage technique used by pronase immunofluorescence, which provides evidence against the glomerular disease of the complex immune type in the setting of MPO-ANCA seropositivity. Despite the withdrawal of hydralazine and prompt initiation of immunosuppressive therapy and alternating sessions of plasmapheresis, the patient succumbed to acute massive pulmonary hemorrhage and subsequent demise. We recommend that patients on the common antihypertensive, hydralazine, should be monitored with non-specific inflammatory markers and, if warranted, with qualitative and quantitative assessment tools to measure inflammatory disease activity for possible complications of hydralazine drug-induced vasculitis or hydralazine ANCA-associated vasculitis (HAAV). Furthermore, cumulative dosages may be a predisposing factor for HAAV to present as a pulmonary-renal syndrome, which can be fulminant and fatal, despite aggressive efforts. Therefore, screening, revisiting therapy, early diagnosis, and prompt discontinuation of the drug are imperative.

## Introduction

Vasculitis is a condition that causes changes in the walls of blood vessels, including thickening, weakening, narrowing, and scarring, resulting in inflammation and necrosis of the blood vessel walls; its clinical features depend on the size, type, and location of the blood vessel that is affected [1,2]. Classification of these vasculitides by vessel size was formulated by the Chapel Hill international consensus conference, which ranges from large-sized vessels like the aorta, to medium-sized vessels like renal and lobar arteries, to small-sized vessels like arcuate and interlobar arteries, the arterioles, and the glomerulus [1,3]. Small-vessel vasculitis is commonly associated with anti-neutrophil cytoplasmic antibodies (ANCA), which activate cytokine-primed neutrophils and monocytes that express ANCA antigens, proteinase 3 (PR3), and myeloperoxidase (MPO) on their surface [3]. The inflammation occurs by ANCA activation of neutrophils, which bind to endothelial cells producing marked expression of adhesion molecules and secretion of pro-inflammatory cytokines including nitric oxide, reactive oxygen species, and proteolytic enzymes [3]. The localized inflammation damages the endothelial cells, which activate MPO to induce detachment, PR3 to cause direct apoptosis, and recruitment of T cells and monocytes [3]. The continuous injury and inflammation of these small vessels characterized by circulating immune complexes and antinuclear antibodies result in clinical features standard in all types of vasculitis, including fever, night sweats, malaise, and arthralgias. In this report, we discuss a rare case of fulminant hydralazine-induced ANCA-associated vasculitis (HAAV) in a patient who was on hydralazine for 10 years.

## Case presentation

**Figure 1:**
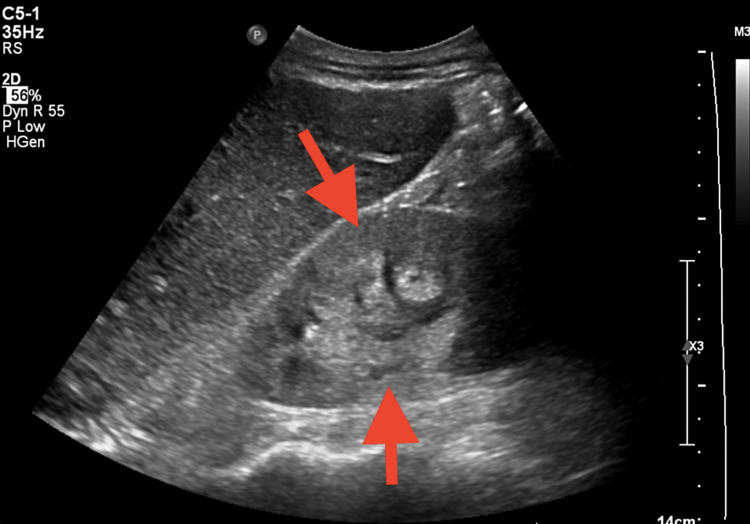
Renal ultrasound showing kidneys with size and parenchymal thickness within normal limits but increased echogenicity, suggesting medical renal disease (arrows) The right and left kidneys measure 10.6 x 5.2 x 4.5 cm and 10.6 x 4.6 x 3.8 cm, respectively. Both kidneys are increased in echogenicity suggesting medical renal disease. No hydronephrosis, shadowing renal calculus, or perinephric fluid collection.

**Figure 2:**
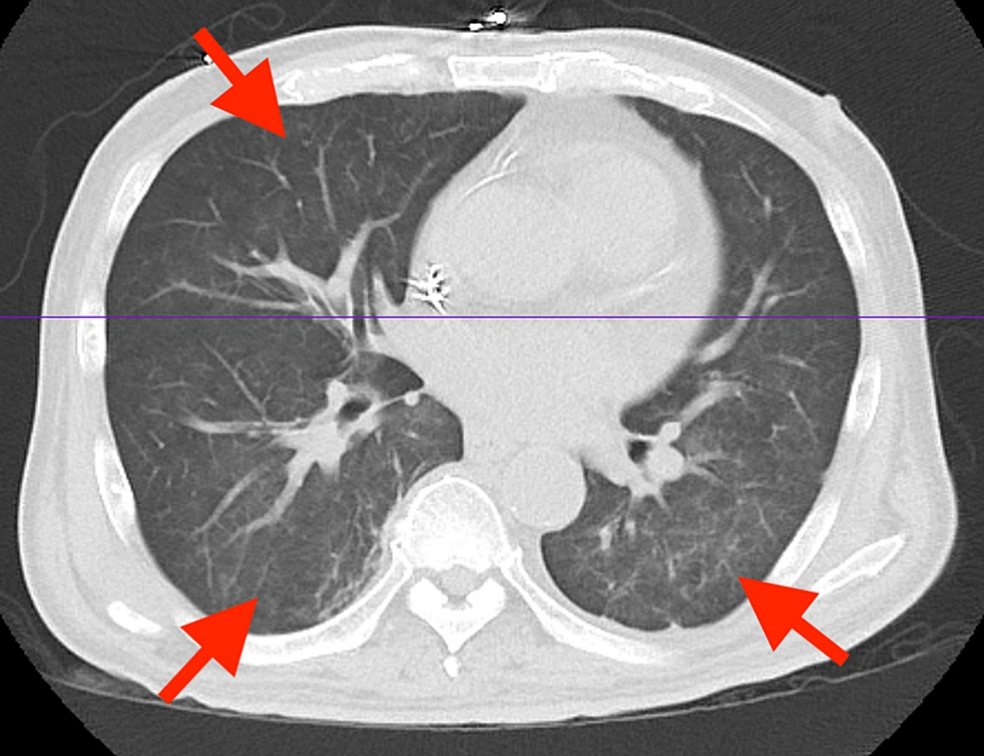
Abdominal and pelvic CT showing mild diffuse bilateral ground-glass opacities, which may reflect infection, inflammation versus pulmonary edema (arrows) CT: computed tomography

**Figure 3:**
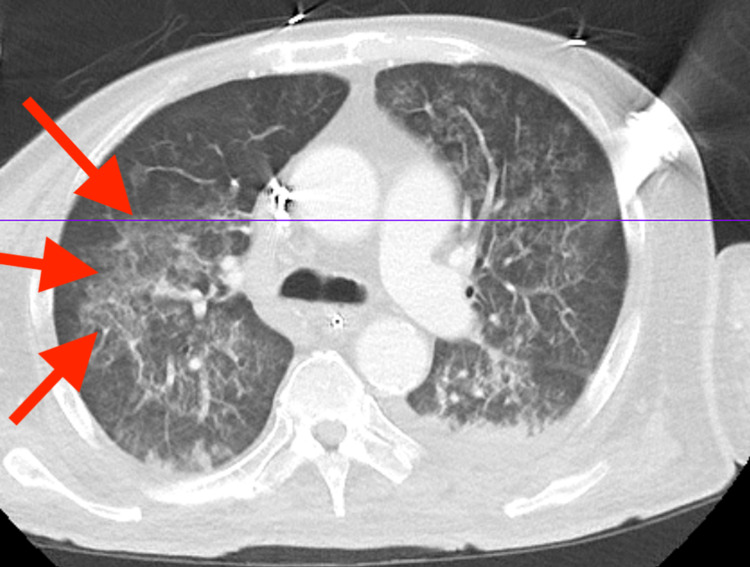
Abdominal and pelvic CT with contrast The image shows large mass-like opacity in the right upper lobe (arrows) with central small foci of air and necrosis that could represent extensive pulmonary consolidation and abscess formation versus mass, and diffuse bilateral ground-glass opacities with confluent areas of consolidation concerning for pneumonia versus pulmonary hemorrhage or edema CT: computed tomography

**Figure 4:**
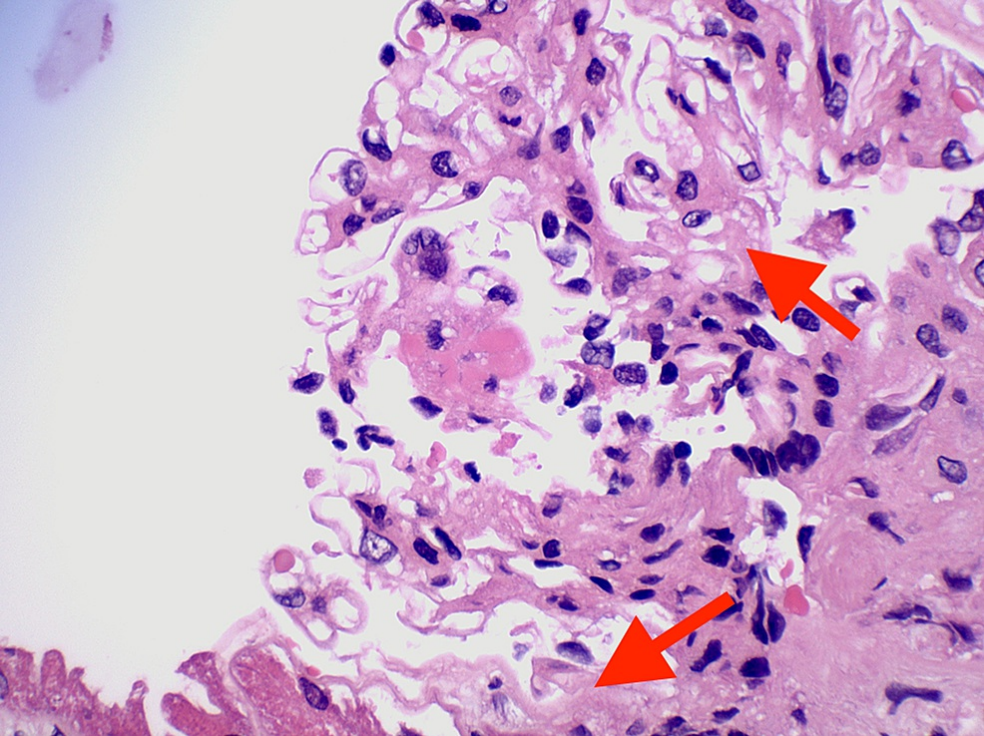
Renal biopsy showing diffuse sclerosing glomerulonephritis with fibrous crescents and a necrotizing lesion, pauci-immune type (MPO-ANCA and hydralazine-associated) (arrows) ANCA: anti-neutrophil cytoplasmic antibody; MPO: myeloperoxidase

**Figure 5:**
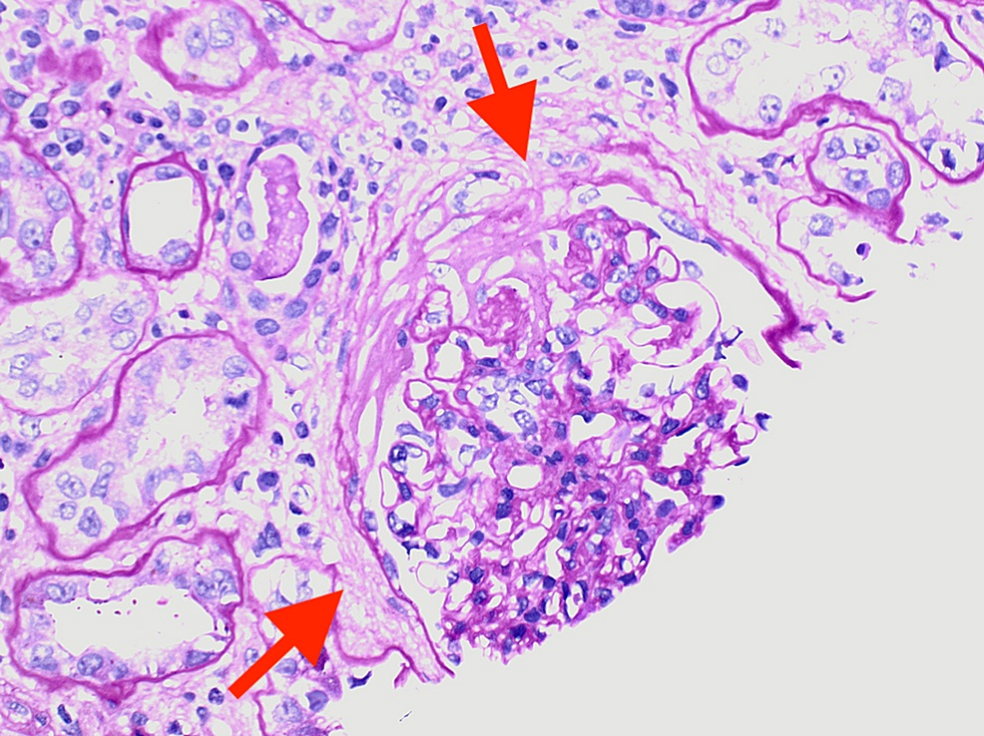
Renal biopsy showing glomerulonephritis exhibiting severe chronicity and focal mild to moderate arterio- and arteriolosclerosis activity (arrows)

**Figure 6:**
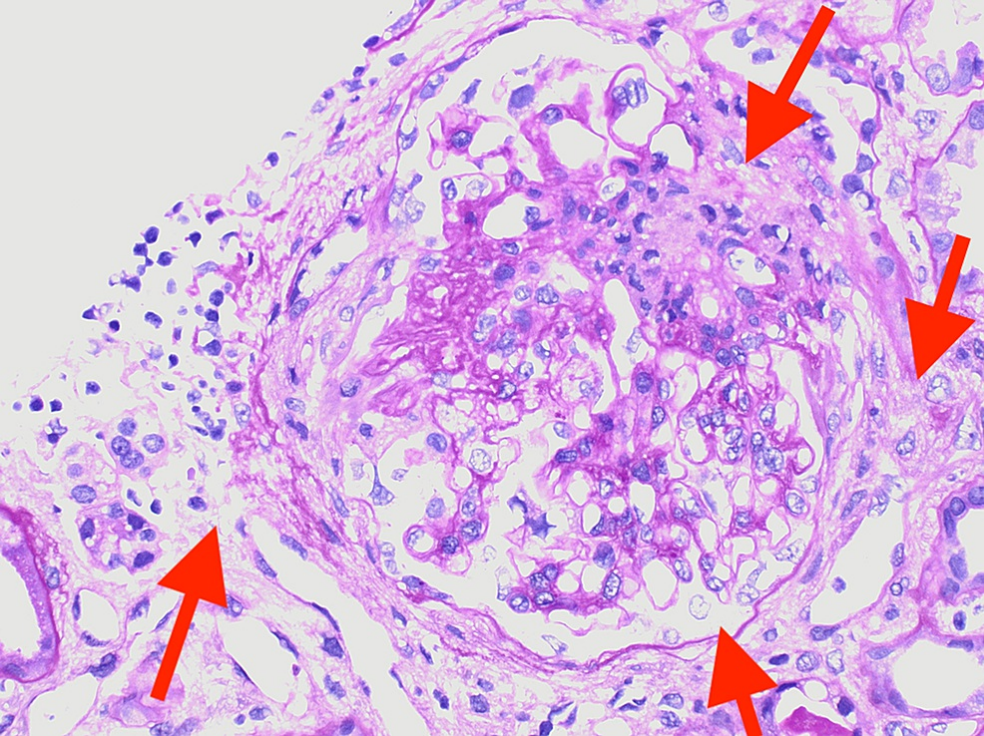
Renal biopsy showing tubular atrophy and severe interstitial fibrosis with moderate interstitial inflammation and patchy tubular degenerative changes (arrows)

**Figure 7:**
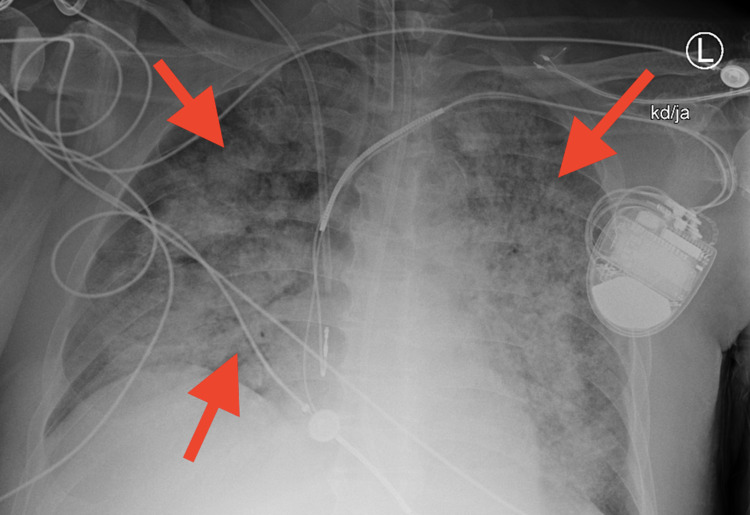
Chest X-ray showing near total atelectasis of the left lung, with interval increased bilateral opacities consistent with pulmonary hemorrhage (arrows)

**Figure 8:**
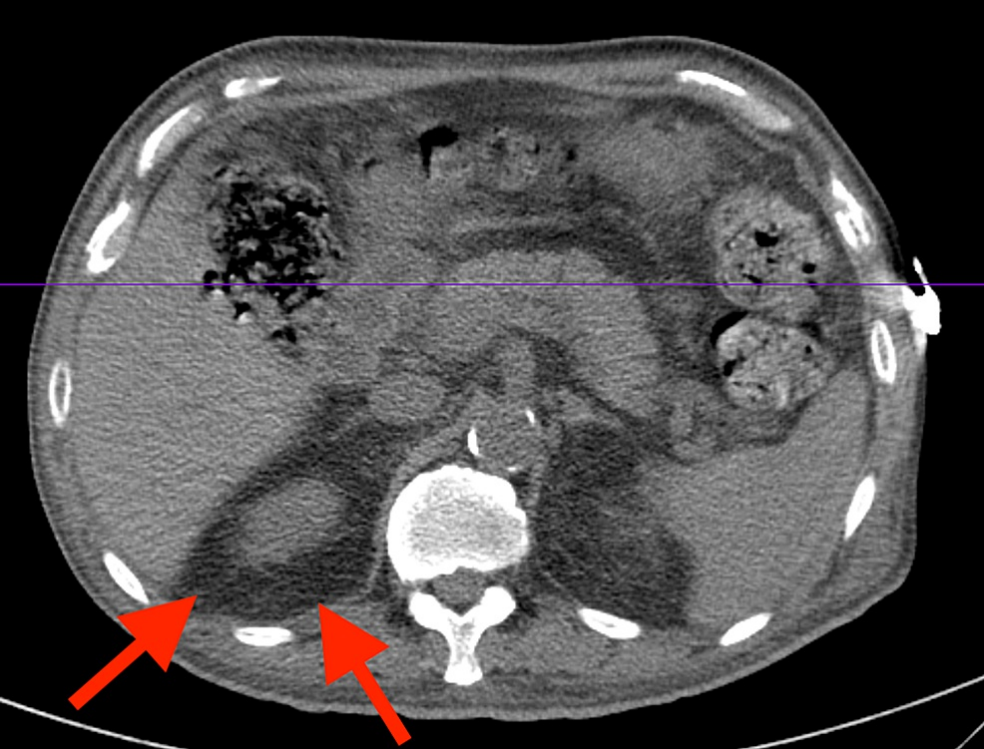
Abdominal and pelvic CT showing right-sided retroperitoneal hemorrhage (arrows) Subject to the imposed limitations, apparent resolution of the duodenal and pancreatic inflammatory changes. Thickened duodenum with mesenteric fat inflammatory changes suggesting duodenitis versus non-perforated peptic ulcer disease. Peripancreatic inflammatory changes are seen around the pancreatic head and are likely reactive; however, primary pancreatitis with reactive thickening of the duodenum cannot be excluded CT: computed tomography

A 59-year-old male with a history of heart failure with a New York Heart Association (NYHA) class III status and an improved ejection fraction of 60% status-post automatic implantable cardioverter-defibrillator (on isosorbide mononitrate 60 mg daily), hypertension [on amlodipine 10 mg and ramipril 10 mg daily, and hydralazine 100 mg every eight hours (for 10 years)], diabetes mellitus (on pioglitazone 30 mg and canagliflozin 300 mg daily, and metformin 1 gm every 12 hrs), and dyslipidemia (on atorvastatin 80 mg and aspirin 81 mg daily) presented with hematuria, intermittent periumbilical abdominal pain, and 40-lb weight loss over four months. The patient had initially visited his local hospital in the Philippines; after receiving three packed red blood cells units, he had signed out against medical advice to travel to the US for further diagnostic workup. The patient presented to the hospital complaining of persistent hematuria and symptomatic anemia. Urine analysis showed large blood cells and proteinuria, which prompted additional studies and suspicion for renal involvement, which was confirmed by imaging (Figure 1). During his clinical course, the patient developed a new diffuse purpuric rash. Systemic involvement was also evidenced by imaging with ground-glass opacities, significant for diffuse alveolar hemorrhage and peripancreatic inflammatory changes, consistent with small-vessel vasculitis (Figures 2, 3). Immunological tests confirmed ANCA-associated vasculitis, and kidney biopsy showed ANCA-mediated pauci-immune glomerulonephritis supported by the salvage technique used by pronase immunofluorescence, which provides evidence against the glomerular disease of the complex immune type in the setting of MPO-ANCA seropositivity (Figures 4, 5, 6). The clinical picture was consistent with HAAV affecting small vessels. In addition to withdrawal of hydralazine, the patient was started on immunosuppressive therapy with methylprednisolone, cyclophosphamide, and rituximab with alternating sessions of plasmapheresis. However, he succumbed to acute massive pulmonary and retroperitoneal hemorrhage and ultimately passed away (Figures 7, 8).

## Discussion

Drug-induced vasculitis is the most common form of vasculitis, and it is associated with MPO-ANCA, PR3-ANCA, elastase, lactoferrin, lysosomal antigens, and autoantibodies to histones and beta-2 glycoprotein I [2,4]. Medications related to drug-induced vasculitis are antibiotics (cefotaxime, minocycline), antithyroid drugs (benzylthiouracil, carbimazole, methimazole, propylthiouracil), anti-tumor necrosis factor-𝛼 agents (adalimumab, etanercept, infliximab), psychoactive agents (clozapine, thioridazine), and other miscellaneous drugs (allopurinol, D-penicillamine, hydralazine, levamisole, phenytoin, sulfasalazine) [2].

Our patient had a history of heart failure with an NYHA class III status and an improved ejection fraction of 60% status-post automatic implantable cardioverter-defibrillator and hypertension optimized with isosorbide mononitrate 60 mg, amlodipine 10 mg, and ramipril 10 mg daily, and hydralazine 100 mg every eight hours for more than 10 years. Hydralazine, an anti-hypertensive medication, acts by inhibiting inositol triphosphate-induced calcium release from the sarcoplasmic reticulum in smooth muscle cells producing vasodilation in resistant arterioles and thereby lowering the blood pressure [5]. Hydralazine is mainly limited to the resistant or intolerant, or standard anti-hypertensive therapy. However, it has been used alongside nitrates in treating African-American patients with NYHA class III and IV heart failure following the ​African-American Heart Failure ​Trial (​A-HeFT) in 2004 [6]. Hydralazine was discontinued in this patient because it has been associated with drug-induced lupus erythematosus, more commonly, as well as ANCA-associated vasculitis, a potentially more serious condition [2].

ANCA-associated vasculitis is the most typical systemic small-vessel vasculitis in adults and has an incidence of 20-30 per million population [7]. A study published in August 2021 identified 80 cases of HAAV complicated by rapidly progressive glomerulonephritis, accounting for 4.3% of ANCA-related glomerulonephritis diagnosed between 2006 and 2019, where over three-fourths of patients were on hydralazine for at least one year, with a mean daily dose of approximately 250 mg/day [8]. Furthermore, HAAV has an incidence of 5.4% in patients on 100 mg/day and 10.4% in those on 200 mg/day for more than three years' duration [9].

Kumar et al. described 323 cases of ANCA-associated vasculitis over 15 years, of which 12 were exposed to hydralazine therapy for an average duration of 22 months and a mean cumulative dose of 146 gm, and concluded that HAAV is commonly associated with pauci-immune glomerulonephritis, interstitial lung disease, and hypocomplementemia [10]. Short and Lockwood, who studied 10 cases of HAAV, suggested that double-stranded DNA, MPO, and lactoferrin autoantibodies against components of the neutrophil cytoplasm are characteristic findings of HAAV [11]. Unlike drug-induced lupus syndrome caused by hydralazine, HAAV patients require immunosuppressive therapy in addition to the withdrawal of hydralazine [10,11]. 

The renal involvement in HAAV is a rare occurrence and is characterized by the lack of immunoglobulins and complement deposition, as demonstrated by a case report of HAAV with pulmonary-renal syndrome [12]. The renal involvement in HAAV is characterized by diffuse sclerosing glomerulonephritis with fibrous crescents (Figure 4), a pauci-immune type seen in MPO-ANCA and hydralazine-associated glomerulonephritis. This diagnosis is supported by the salvage technique used by pronase immunofluorescence, which provides evidence against the glomerular disease of the complex immune type in the setting of MPO-ANCA seropositivity. The glomerulonephritis exhibits severe chronicity and mild focal activity demonstrated by moderate arterio- and arteriolosclerosis (Figure 5). In pauci-immune type crescentic glomerulonephritis, as seen in MPO-ANCA and HAAV, there may be tubular atrophy and interstitial fibrosis, ranging from mild to severe, as well as mild to moderate interstitial inflammation and patchy tubular degenerative changes (Figure 6). This autoimmune phenomenon is characteristic of ANCA-mediated glomerulonephritis in the setting of hydralazine. The pathogenesis of hydralazine-induced vasculitis is not well understood. Still, one theory states that hydralazine accumulates in neutrophils, binds to myeloperoxidase, and induces cytotoxic products and neutrophil apoptosis [13]. Differentiating idiopathic ANCA vasculitis from hydralazine-induced vasculitis is difficult, but treatment after discontinuing the suspected drugs is essentially the same, including steroids and cytotoxic therapy [14]. With HAAV, up to 37% of patients may reach the combined endpoint of end-stage renal disease (ESRD) or death, with the remaining 63% associated with persistent ANCA positivity despite discontinuing hydralazine and inducing immunosuppression as demonstrated in a study of 80 cases of HAAV complicated by rapidly progressive glomerulonephritis discontinuation, which was published in August 2021 [8]. HAAV presenting as a pulmonary-renal syndrome can be fulminant and fatal, despite aggressive efforts to manage it [12].

Regular monitoring of specific inflammatory markers may reveal an underlying condition, and it only requires a small amount of blood. These inflammatory markers, erythrocyte sedimentation rate (ESR) and C-reactive protein (CRP), could be collected with the same blood sample used for standard annual laboratory tests. Persistent low-grade inflammation can be evidenced by elevated ESR and CRP levels. Both CRP and ESR yield non-specific information about inflammation, but one notable difference between the two tests is that changes are reflected more quickly with CRP compared to ESR [15]. For example, CRP levels may drop to normal levels following successful treatment of an infection more quickly, while ESR remains elevated for a more extended period [15].

Cost-effectiveness is an essential factor to be considered when conducting regular screening of blood tests when there is no evidence of disease. The CRP test is inexpensive (around $12 to $16), whereas ESR is costlier (around $26 to $60), and with health insurance, these tests should be covered with only a small co-pay, as compared to ANCA testing (above $120) [16]. ESR and CRP are non-specific tests that may be elevated in a number of different conditions but provide general information about the presence or absence of inflammatory disease. When screening becomes a costly burden, there are other ways to measure inflammatory activity without resorting to expensive antibody testing that can be used when non-specific inflammatory markers are elevated. Providers may use non-expensive qualitative and quantitative tools to assess and measure inflammatory disease activity, thereby providing the clinical basis for decision making regarding therapy, long-term patient care, and determining prognosis or outcome.

**Figure 9:**
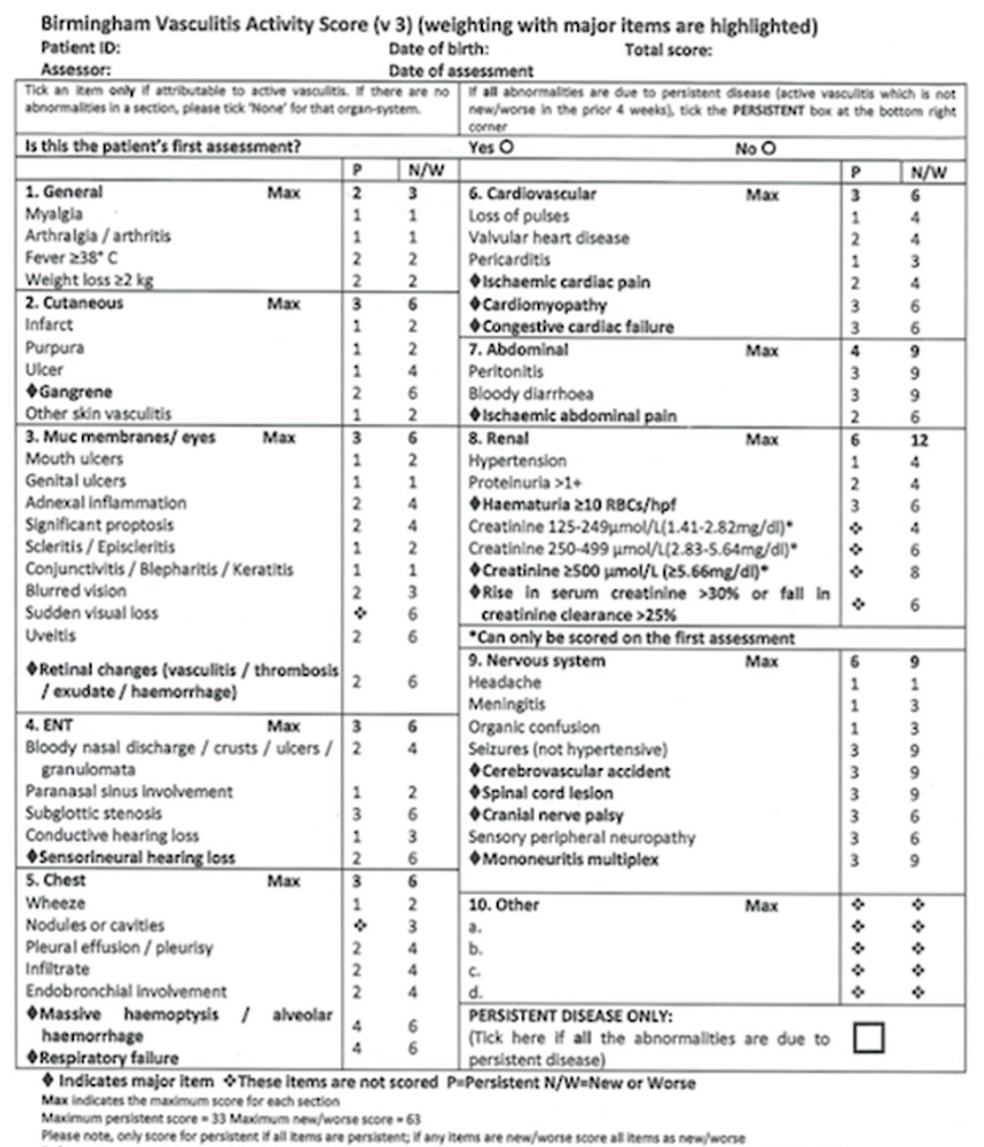
Birmingham Vasculitis Activity Score (Version 3) Only those disease features attributed to active vasculitis are scored, after excluding other causes, such as infection, hypertension, etc. If a new feature has occurred or if there has been a recent deterioration of status since the last visit, it is scored in the New/Worse box. If abnormality indicates the presence of active vasculitis (but not New or Worse), the Persistent box is ticked. The data score sheet will be used to derive indices of disease activity as follows: -BVAS.1 (New/Worse): represents a score of New/Worse activity attributable to vasculitis -BVAS.2 (Persistent): represents a score of disease activity due to persisting disease, which is neither new nor worse than the previous assessment If the patient scores over the maximum number in a system, they only score the maximum score stipulated. Add the scores for each system together to get the final score There are two separate final scores: BVAS New/Worse - max score of 63 BVAS Persistent - max score of 33 The higher the score, the more active the disease

**Figure 10:**
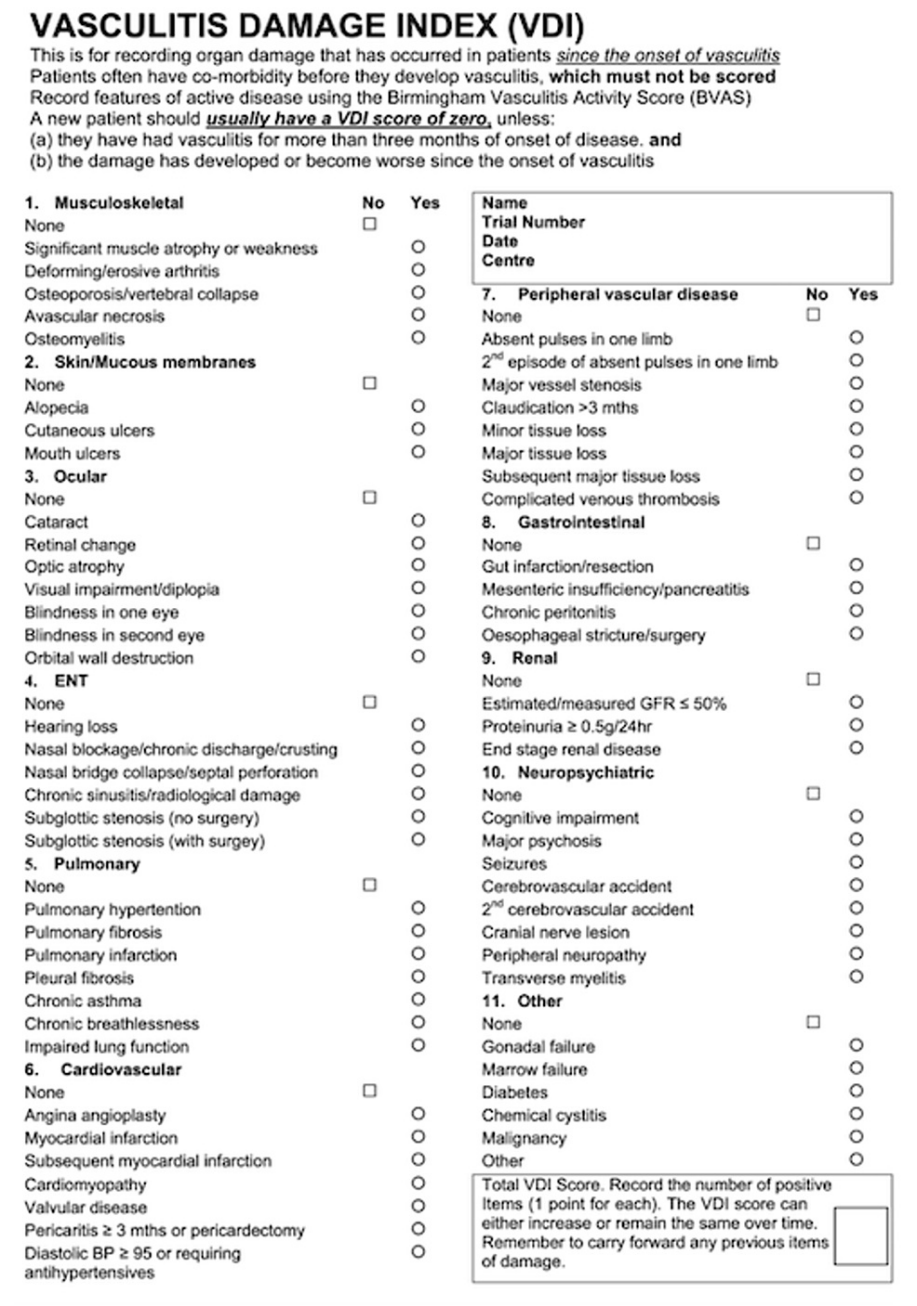
Vasculitis Damage Index Disease features that have occurred since the onset of vasculitis are scored, regardless of whether or not they are attributable to vasculitis. It is straightforward to calculate; each item contributes 1 point to the total score. However, it is essential to remember that it is cumulative. Therefore, each time VDI is evaluated, you should include all the items from the previous assessment and add any additional ones (i.e., it never gets better). The scoring sheet is divided into 10 systems plus the 11th section for other items, mainly related to the effects of drugs. However, there is one space left for an “other” item thought to be representative of damage, which has not already been recorded elsewhere on the VDI form. Each item scores 1 point. Therefore, we can calculate three possible scores for each patient: Total VDI scores - the total number of items scored: min score of 0, max score of 64 System score - the extent of disease defined by the number of separate systems with at least one item scored Critical damage score - the number of items of damage consistent with organ failure Add up the number of positive items. The VDI score can increase or remain the same over time. When patients present for the first time and their symptoms have only been present for less than three months, the VDI score is automatically zero. However, if patients have suffered a specific item of damage within those three months or have continued to suffer for more than three months, then at the following VDI assessment, you will need to record that as a positive item. If you are seeing a patient for the first time, with an onset of features of vasculitis lasting more than three months, some damage items may be recorded at baseline

There are many tools to measure disease activity in vasculitis, but due to multi-system involvement, any clinical assessment requires evaluation in each body system [17]. The Birmingham Vasculitis Activity Score (BVAS) is a validated tool in evaluating disease activity in patients with many different forms of vasculitis [17,18]. The BVAS includes scored items grouped into nine organ systems that capture a broad spectrum of clinical manifestations of vasculitis (Figure 9) [19], [17-20]. Only those features attributed to active vasculitis are considered [17,18]. The Vasculitis Damage Index (VDI) is divided into 11 organ systems and records damaged items due to vasculitis, treatment, or unrelated, which have occurred since the onset of vasculitis (Figure 10) [19], [17-20]. Nevertheless, BVAS and VDI are internationally validated and recognized assessment tools that enable the comparison of studies from different continents and effective collaboration in multi-center studies [17,18,20].

Once there is evidence of a possible vasculitis, ANCA testing with MPO and PR3 antibodies can be pursued to establish or rule out a diagnosis. After establishing HAAV diagnosis, the goal of induction therapy is the rapid, effective suppression of the immune response to limit inflammatory organ injury [8]. Additionally, maintenance therapy provides lower intensity immunosuppression over the medium to long term to prevent disease relapse and organ damage [8].

## Conclusions

Hydralazine remains a common antihypertensive medication for the management of hypertension. However, due to the possible complications of hydralazine drug-induced vasculitis, we recommend that non-specific inflammatory markers, ESR and CRP, be regularly monitored in patients using this drug. Furthermore, early detection of an underlying inflammatory condition could facilitate the use of non-expensive qualitative and quantitative assessment and measurement tools of inflammatory disease activity, such as BVAS and VDI, providing a clinical basis to test for ANCA, MPO, and PR3 antibodies, and thereby prompting the provider to discontinue hydralazine and further investigate an underlying vasculitis. However, given the low incidence of HAAV, additional blood tests at regular check-ups may be a costly burden. Of note, our patient was on hydralazine for 10 years, and cumulative dosage was a possible predisposing factor. Hence, revisiting therapy, early diagnosis, and prompt discontinuation of the drug are imperative.
